# COVID-19 Health Beliefs Regarding Mask Wearing and Vaccinations on Twitter: Deep Learning Approach

**DOI:** 10.2196/37861

**Published:** 2022-10-31

**Authors:** Si Yang Ke, E Shannon Neeley-Tass, Michael Barnes, Carl L Hanson, Christophe Giraud-Carrier, Quinn Snell

**Affiliations:** 1 Department of Statistics Brigham Young University Provo, UT United States; 2 Department of Public Health Brigham Young University Provo, UT United States; 3 Computer Science Department Brigham Young University Provo, UT United States

**Keywords:** COVID-19, Health Belief Model, deep learning, mask, vaccination, machine learning, vaccine data set, Twitter, content analysis, infodemic, infodemiology, misinformation, health belief

## Abstract

**Background:**

Amid the global COVID-19 pandemic, a worldwide infodemic also emerged with large amounts of COVID-19–related information and misinformation spreading through social media channels. Various organizations, including the World Health Organization (WHO) and the Centers for Disease Control and Prevention (CDC), and other prominent individuals issued high-profile advice on preventing the further spread of COVID-19.

**Objective:**

The purpose of this study is to leverage machine learning and Twitter data from the pandemic period to explore health beliefs regarding mask wearing and vaccines and the influence of high-profile cues to action.

**Methods:**

A total of 646,885,238 COVID-19–related English tweets were filtered, creating a mask-wearing data set and a vaccine data set. Researchers manually categorized a training sample of 3500 tweets for each data set according to their relevance to Health Belief Model (HBM) constructs and used coded tweets to train machine learning models for classifying each tweet in the data sets.

**Results:**

In total, 5 models were trained for both the mask-related and vaccine-related data sets using the XLNet transformer model, with each model achieving at least 81% classification accuracy. Health beliefs regarding perceived benefits and barriers were most pronounced for both mask wearing and immunization; however, the strength of those beliefs appeared to vary in response to high-profile cues to action.

**Conclusions:**

During both the COVID-19 pandemic and the infodemic, health beliefs related to perceived benefits and barriers observed through Twitter using a big data machine learning approach varied over time and in response to high-profile cues to action from prominent organizations and individuals.

## Introduction

On January 30, 2020, the World Health Organization (WHO) declared the Chinese outbreak of SARS-CoV-2 (ie, COVID-19) to be a public health emergency of international concern [1]. The following day, the United States Department of Health and Human Services (HHS) secretary declared a US public health emergency to respond to COVID-19 [1]. The president of the United States signed a “Proclamation on Suspension of Entry as Immigrants and Nonimmigrants of Persons Who Pose a Risk of Transmitting 2019 Novel Coronavirus,” limiting entry into the United States of persons who traveled to mainland China. Subsequently, on March 11, 2020, WHO declared COVID-19 to be a global pandemic.

With the emergence of a global pandemic came another concern, the emergence of a worldwide infodemic. As it pertained to COVID-19, WHO described the infodemic as an overabundance of information and misinformation related to the COVID-19 pandemic that led to mistrust of health authorities and hampered public health efforts [2]. With the growth in social media use, information about COVID-19 spread quickly, necessitating infodemic management or the need to manage false and misleading information in such a way that would reduce the impact on health behaviors [2]. Greater attention is being paid to sources of COVID-19 information, especially low-credibility sources responsible for spreading COVID-19 misinformation through social media channels [3]. As such, several researchers have begun to address methods for fighting the COVID-19 infodemic and acknowledge the influential role of social media [4-7]. The continuous monitoring and analysis of social media information (infodemiology) has been heralded as a critical tool for understanding the influence of social media and combating misinformation [5]. Although social media data are not specifically designed for public health purposes, they are a valuable and accessible resource for public health surveillance purposes [8]. For example, topic modeling of Twitter posts has been used in understanding topics and sentiments related to COVID-19 in general [9-11], face masks [12,13], and vaccine discussions [14]. Although simply monitoring social media information can provide valuable insight into COVID-19 information/misinformation, understanding the influence on health beliefs and behaviors during an infodemic can assist public health in better managing information during health emergencies, such as COVID-19, through risk communication [14]. In addition, less is known about the influence of higher-credibility sources of information, such as prevention guidelines coming from WHO and the Centers for Disease Control and Prevention (CDC).

**Figure 1:**
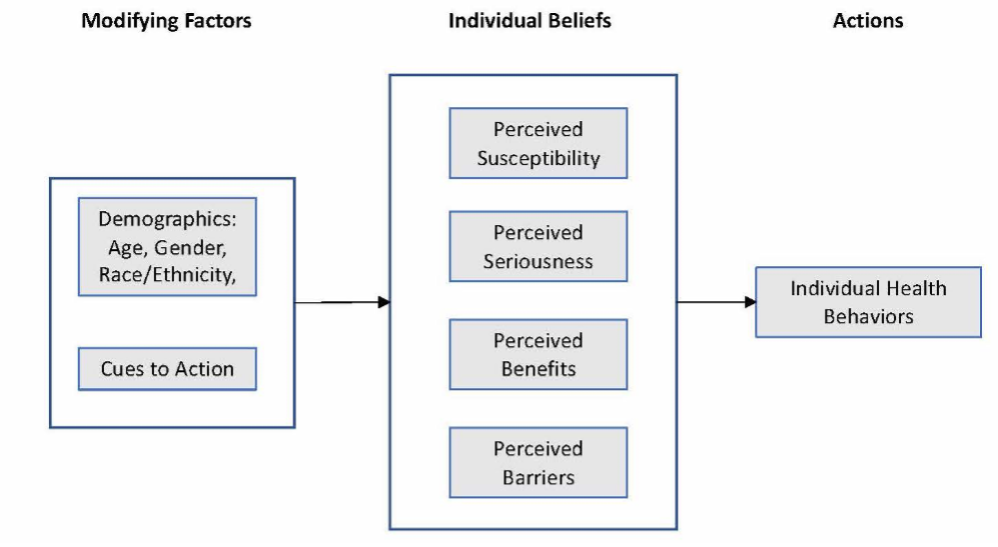
Health Belief Model (HBM).

The Health Belief Model (HBM) was developed to explain how beliefs impact health decisions [15]. The theory posits that people engage in health-related behaviors based on (1) their perception of the health condition (eg, COVID-19), (2) their perception of the advantages and disadvantages of the health behavior (eg, mask wearing or receiving a vaccine), and (3) cues to action or stimuli that encourage them to participate in the behavior (eg, health organization recommendations). This theory consists of 5 main elements: perceived susceptibility, perceived severity, perceived benefits, perceived barriers, and cues to action (Figure 1). The model has been successfully used to assess health beliefs on social media regarding physical distancing during the COVID-19 pandemic [16], Zika virus [17], and the human papillomavirus vaccine [18]. Although traditional polling methods require a substantial number of resources and have limitations in assessing public health beliefs (eg, difficulty reaching a large-scale population in large geographic areas and tracking changes in real time), social media has provided millions of people, worldwide, a chance to voluntarily and continuously express their thoughts and opinions on issues that they deem important [18]. Although 1 study used machine learning of Twitter posts to monitor health beliefs regarding COVID-19, health care treatments, and the influence of various external cues to action [19], no identified study has used this methodology to explore the HBM regarding important COVID-19–related behavioral outcomes—mask wearing and vaccinations.

Research has demonstrated that health organizations, physicians, and the media during COVID-19 represent important HBM cues to action [20]. WHO and the CDC have both issued COVID-19 prevention recommendations; however, recommendations have evolved over time. For example, in the first official advice document regarding the need for and usage of masks, WHO stated that a medical mask is not required for healthy individuals, as no evidence was available on its usefulness to protect nonsick persons [21]. Later, in the updated mask guidelines, the previous advice was modified, and the general public was encouraged to wear masks [22]. Similarly, the CDC initially asserted that the wearing of face masks was an unnecessary public health tool, but a short while later, it issued new guidelines advising people to wear face coverings in public settings where social distancing was difficult [23]. Understanding the individual beliefs regarding COVID-19–preventive behaviors in response to various cues to action from these high-credibility sources is crucial toward helping manage an infodemic. The fallout from these types of COVID-19 shifts in prevention guidelines have created controversy among many sources.

Generally, US guidelines emerging from national, state, and local public health organizations received prominent attention. Lessons have been learned from global and national guidelines, including the following: (1) Travel restriction delays allowed citizens traveling from high-risk areas to pass freely through airports without screening; (2) quarantine delays in high-risk areas allowed potentially infected individuals to spread the infection; (3) public misinformation allowed racism, incorrect public precautions, and unprecedented fear surrounding COVID-19, allowing rumors, speculation, and misinformation to spread; and (4) emergency announcement regarding the outbreak severity was delayed and not widely broadcast for a month when WHO declared the public health emergency of international concern [24]. Additionally, the WHO guidelines came under political scrutiny by the US president, who blamed WHO for delays and dysfunctions to investigate early cases of COVID-19 and suspended WHO funding [25].

The purpose of this study is to investigate health beliefs and cues to action for mask wearing and vaccination using machine learning of COVID-19–related Twitter posts. External cues to action from prominent pandemic declarations (eg, WHO and the CDC) regarding mask wearing and vaccines and prominent examples of displayed preventive behaviors (eg, presidential mask wearing) were explored for possible influence on health beliefs, as explained by HBM constructs. Although these prominent events could not be studied for a cause-effect relationship, given the surveillance approach of this study, observing the prominent events along with the ongoing Twitter posts may help provide clues to their potential effect on cues to action. This unique approach is an important way to begin exploring how infodemics may influence cues to action. Tables 1 and 2 show the HBM constructs for masks and vaccines, respectively. Findings from this study revealed that cues to action are associated with increased conversations around the perceived health beliefs about mask wearing and vaccinations.

**Table 1:** HBM^a^ constructs related to COVID-19 and face coverings.

Construct	Definition
Perceived susceptibility	Assessment of the likelihood or risk of contracting COVID-19; increased likelihood of contracting the disease (eg, increased/decreased prevalence, high/low number of COVID-19 cases)
Perceived severity	Assessment of the perceived seriousness and consequences of contracting COVID-19 (eg, hospitalization, death, mortality, disability)
Perceived benefits	Comments mentioning the benefits of masks or face coverings to reduce the transmission of COVID-19 or the removal of barriers (eg, promotion of mask or face coverings)
Perceived barriers	Comments mentioning the difficulties, challenges, and negative effects of masks and face coverings or the perceived ineffectiveness of masks and face coverings (eg, negative reports of masks or face coverings)

^a^HBM: Health Belief Model.

**Table 2:** HBM^a^ constructs related to COVID-19 and vaccines.

Construct	Definition
Perceived susceptibility	Assessment of one’s likelihood or risk of contracting COVID-19 if not vaccinated; references increased/decreased prevalence, high/low number of cases, and high/low risk/chance/probability
Perceived severity	Assessment of the seriousness of COVID-19 and the major consequences that contracting COVID-19 would have on one’s life, such as hospitalization, death, mortality, or disability
Perceived benefits	Assessment of the benefits of COVID-19 vaccines or being vaccinated against COVID-19; the removal of barriers (see the Perceived Barriers section for more information); positive opinion
Perceived barriers	Assessment of the barriers to COVID-19 vaccination, including difficulties, challenges, conspiracies, negative effects, dangers, and perceived ineffectiveness; the removal of benefits; negative opinion

^a^HBM: Health Belief Model.

## Methods

### Data Collection

For this paper, a large, publicly available data set of COVID-19–related tweets was used [26,27]. Since Twitter’s terms of service only allow the tweet IDs to be publicly available, the authors hydrated the tweet IDs with their own Twitter developer accounts.

**Figure 2:**
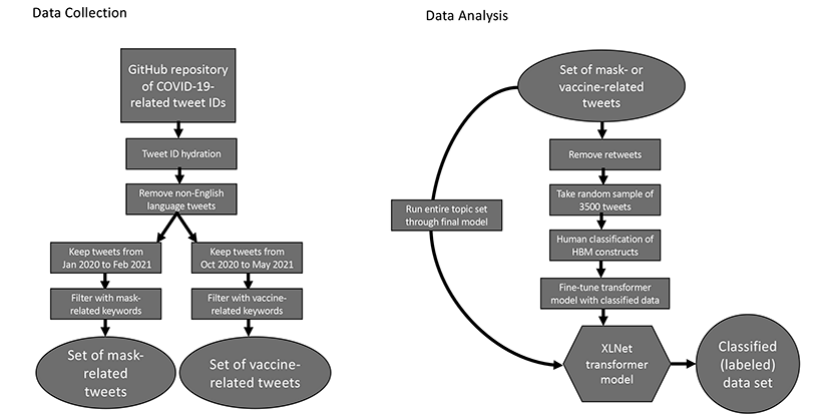
Data collection and analysis procedures. HBM: Health Belief Model.

The diagram on the left in Figure 2 outlines the data collection process. Since the transformer models used are pretrained and can handle rather raw information, only minor preprocessing of the data was necessary. Non-English tweets were excluded, and all text was converted to lowercase. Tweets were then filtered by date, and an iterative process was used to filter the tweets by keywords. Research students came up with initial lists of keywords for both mask-related and vaccine-related tweets. The lists were reviewed by the extended research group and modified, as appropriate. For example, the first list included the keyword “face,” which picked up a lot of tweets talking about “Facebook.” Hence, the keyword “face” was changed to “face “ (with a space after the “e”). The keyword lists may not be perfect and may cause the inclusion of tweets outside of the scope of face masks or vaccines related to COVID-19. However, they do produce a more relevant population than the entire corpus, and together with the hand labeling, it was felt that the model would be able to identify HBM-related tweets.

The set of mask-related tweets was created by filtering tweets from January 2020 through January 2021 with the following keywords: “mask,” “face cover,” “facemask,” “cloth cover,” “cover your face,” “face covering,” “maskup,” and “face.” Likewise, the set of vaccine-related tweets was created by filtering tweets from October 2020 through November 2021 with the following keywords: “vaccine,” “antiva,” “anti-va,” “vax,” “shot,” “inoculat,” “needle,” “booster,” “pfizer,” “biontech,” “immun,” “mrna,” “trials,” “moderna,” “novacax,” “astrazeneca,” “johnson,” “sanofi,” and “glaxosmithkline.” As expected, vaccine-related Twitter conversation came later than mask-related conversation, which is reflected in the different start dates of the data collection. As to the end dates, their selection was simply a matter of choice based on the authors’ hypotheses. Upon examination of the mask-related data and resultant graphs, the hypothesized correlations were clearly exhibited, and it was felt that additional data would not significantly change these results (the HBM is all about beliefs affecting behavior), so data collection for the mask-related tweets was interrupted (similarly for the vaccine-related tweets, but for the relevant, later date range).

The final data set statistics are as follows: (1) 1.8 TB on disk, (2) 646,885,238 total tweets, (3) 59,724,507 mask-related tweets, and (4) 113,542,400 vaccine-related tweets.

### Data Analysis

Once the topic-related tweets sets were created, they were classified according to the HBM constructs. The classification process was performed separately for each topic-related set, but the process was the same. A random sample of 3500 tweets was selected for manual labeling. No retweets were included in these sets to avoid biasing the models, since retweets would cause repetition of content. Hence, the labeled data consisted of unique tweets, and only these were used in the subsequent model-building phase. Three independent reviewers manually classified the sample according to their relevance to the HBM constructs. Every tweet in the sample was classified with either a positive or a negative label for each of the 4 HBM constructs (ie, perceived susceptibility, perceived severity, perceived benefits, and perceived barriers). Next, a tweet that was labeled positive for at least 1 of the 4 constructs was also classified as HBM related. Tables 1 and 2 show the criteria for a positive label for each of the 4 HBM constructs for the mask- and vaccine-related tweets, respectively. These criteria were defined using constructs from similar work. Compiled together, each construct is assumed to impact the likelihood for persons taking (or not taking) action. The reviewers classified the tweets independently but compared classifications together after labeling the first 100 tweets and again after the first 500 tweets to resolve conflicts. During these calibration meetings, the reviewers came together and examined samples of tweets that differed in classifications and came to consensus based on the criteria defined in Tables 1 and 2. The final label for each tweet was the label that received the majority vote (ie, at least 2 votes out of 3 from the reviewers).

Note that each tweet could be labeled as belonging to more than 1 of the 4 HBM constructs as 1 part of a tweet could fall into 1 HBM category and another part of the tweet could fall into a different HBM category. As an example, consider the following tweet from the set of mask-related tweets:

Buying reusable masks is generally a waste of time and hurts healthcare workers (hello) who need to use them in everyday care during the flu season. Is the corona virus scary? The weirdo incubation time and severity/rapid spread is honestly wild.

This tweet was labeled for both perceived barriers and perceived susceptibility because the user first advocated not buying reusable masks (perceived barriers) and then proceeded to comment on how fast the virus spreads (perceived susceptibility).

Once labeled, each set of 3500 tweets (mask related and vaccine related) could be used for model-building purposes. A random stratified 2450/1050 (70%/30%) split was applied to create the training and test sets. Each model was trained exclusively on the training data (n=2450, 70%). The only hyperparameter considered was the dropout rate, which did not significantly change the results. The test data (n=1050, 30%) were then used to assess the quality of the models, and the results presented here are based on those data alone. Following their construction, the models were applied to label all tweets, as a real system would indeed be expected to label both original tweets as well as all retweets. The diagram on the right of Figure 2 illustrates the steps taken to process the data from the GitHub repository through the creation of 2 topic-related tweet sets and finally through the classification process.

State-of-the-art bidirectional transformer models [28] were used, combined with custom classification layers. Three different pretrained transformer models were considered and fine-tuned: bidirectional encoder representations from transformers (BERT) [29], a distilled version of bidirectional encoder representations from transformers (DistilBERT; more memory efficient), and XLNet [30]. Three additional simpler models were also included for comparison, namely logistic regression, RepresentationNet (a vanilla version of BERT with a custom classification network), and a bidirectional gated recurrent unit (BiGRU) network [31]. All models were implemented in Python with the *pytorch* library. Each model’s predictive ability was evaluated by the area under the receiver operating characteristic (AUROC) curve, accuracy, precision, recall, and *F*_1_ score. In addition, the final model size (pytorch binary format) and a measure of performance in the form of the number of tweets the model can classify per second were also computed.

After classifying all the tweets using the transformer model, the tweets were separated into calendar weeks and counted. HBM-positive label percentages were computed by taking raw counts of HBM-positive labels divided by the total raw count of COVID-19–related tweets filtered for mask- or vaccine-related keywords, respectively. Potential linear relationships between HBM label percentages by week and COVID-19–related statistics from the corresponding weeks, such as US confirmed case counts, US COVID-19 death counts, and US COVID-19 vaccine doses administered, were also investigated using scatter plots, Spearman correlation matrices, simple linear regression models, and regression with added quadratic terms.

### Ethical Considerations

Ethical approval was not needed, since the study only analyzed publicly available data from existing data sets, and results do not contain any identifiable information and are represented in aggregate only.

## Results

### Manual Labeling and Interrater Reliability

For the mask-related data set, interrater reliability coefficients, measured by Gwet AC1, were 0.784, 0.762, 0.957, and 0.938 for perceived benefits, perceived barriers, perceived severity, and perceived susceptibility, respectively. Interrater reliability coefficients for the vaccine-related data set, measured by Gwet AC1, were 0.825, 0.814, 0.937, and 0.915, respectively, for the 4 HBM constructs in the same order as above. These interrater reliability coefficients are all interpreted as substantial agreement to almost perfect agreement according to the Landis-Koch benchmarking scale [32]. Compared to other interrater reliability calculations, Gwet AC1 is more stable than traditional κ coefficient calculations [33].

For the mask-related data set, 2135 (61%) tweets were manually labeled as related to the HBM model. For the vaccine-related data set, 1330 (38%) tweets were labeled as related to the HBM model.

### Machine Learning Model

Table 3 reports on the AUROC, accuracy, precision, recall, and *F*_1_ score for each model. It also includes the model size, as well as the number of tweets the model can classify per second. All 3 pretrained transformer models outperformed the simpler models. Among the transformer models, the XLNet transformer model (with a custom dense 3-layer classification network and a dropout rate set to 0.25) was clearly the best model for this task.

**Figure 3:**
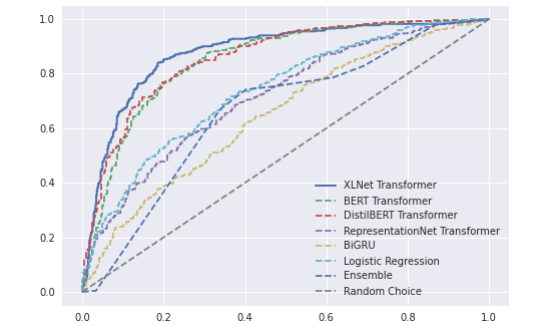
AUROC curves. AUROC: area under the receiver operating characteristic; BERT: bidirectional encoder representations from transformers; BiGRU: bidirectional gated recurrent unit; DistilBERT: distilled version of bidirectional encoder representations from transformers.

To provide a finer-grained analysis of performance, Figure 3 displays the complete AUROC curve for each of the models. The graph confirms the superiority of the transformer models. Furthermore, it shows that the XLNet transformer generally dominates, and at worse is on par with, the other transformer models.

**Table 3:** Model evaluation metrics.

Model	AUROC^a^	Accuracy	Precision	Recall	*F*_1_ score	Size (MB)	Evaluations/second
XLNet	0.878	0.824	0.775	0.761	0.768	467	234.8
BERT^b^	0.850	0.786	0.685	0.760	0.721	440	321.1
DistilBERT^c^	0.858	0.795	0.760	0.692	0.725	261	563.5
RepresentationNet	0.717	0.735	0.644	0.648	0.646	484	316.0
BiGRU^d^	0.737	0.752	0.665	0.678	0.672	27	473.9
Logistic regression	0.566	0.651	0.584	0.229	0.329	0.0016	15,925.3

^a^AUROC: area under the receiver operating characteristic.

^b^BERT: bidirectional encoder representations from transformers.

^c^DistilBERT: distilled version of bidirectional encoder representations from transformers.

^d^BiGRU: bidirectional gated recurrent unit.

It is worth noting the strength of transformer models, especially pretrained ones. Indeed, it is quite remarkable that XLNet achieved over 82% accuracy with only 3500 training data points. By contrast, logistic regression achieved only about 65% accuracy. It is likely that a larger training corpus would improve the performance of logistic regression, but transformer models can do rather well with relatively small corpora. It is also of note that although XLNet is better across predictive evaluation metrics, its memory footprint is significantly larger, and its speed of execution is slower. In cases of low memory, the DistilBERT model may be a viable alternative at the slight cost of some performance loss in classification. Here, the XLNet model was preferred because it fit well within available memory constraints.

Using the XLNet transformer model, 5 models were trained to classify the tweets for HBM relatedness and for the 4 HBM constructs (in the order of perceived benefits, perceived barriers, perceived severity, and perceived susceptibility). The 5 models each achieved over 81% classification accuracy (81%, 97%, 96%, 85%, 82%, respectively) on test data for the mask-related tweets and over 79% classification accuracy (82%, 81%, 86%, 79%, 85%, respectively) on test data for the vaccine-related tweets. Again, the pretrained transformers proved effective at embedding the tweets, making it simple to train the custom classification networks.

**Figure 4:**
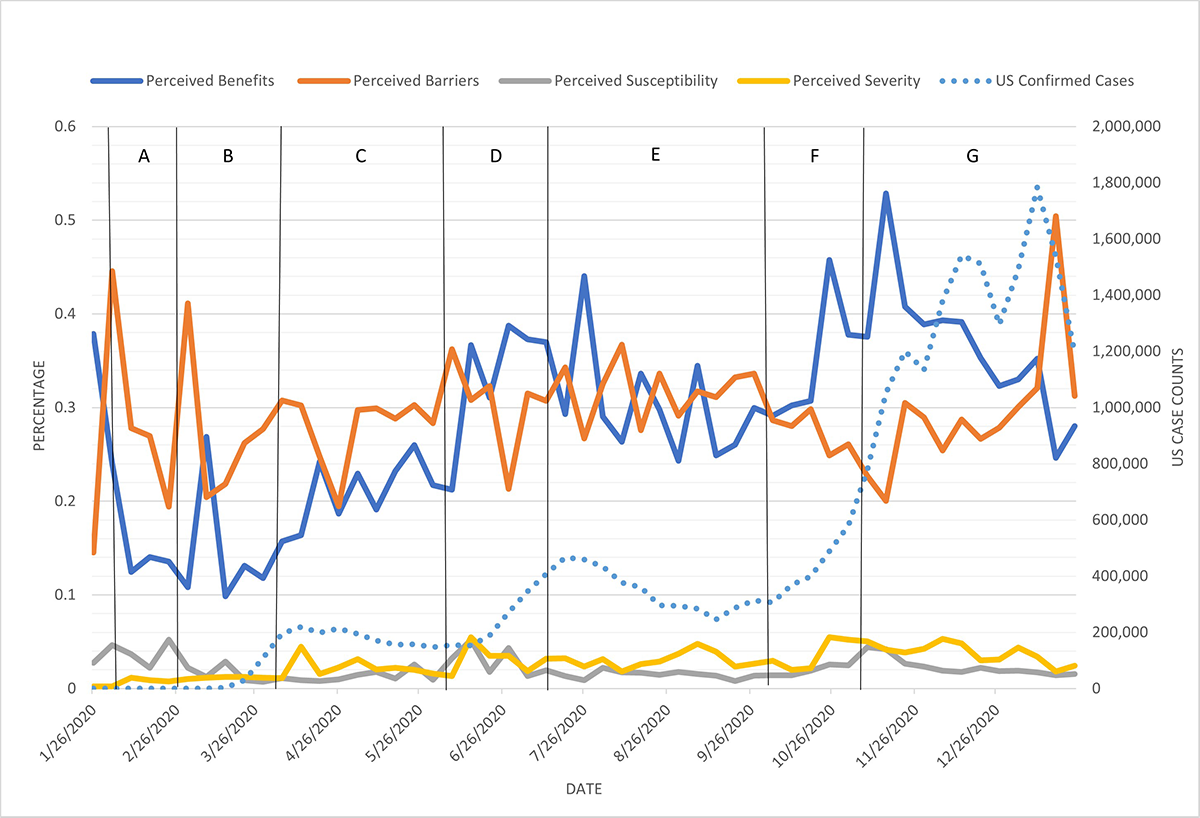
US COVID-19 case counts and each HBM scale across time for mask-related tweets. There was a statistically significant correlation between case counts and perceived benefits. Important events corresponding to the time intervals are listed in Table 4. HBM: Health Belief Model.

**Figure 5:**
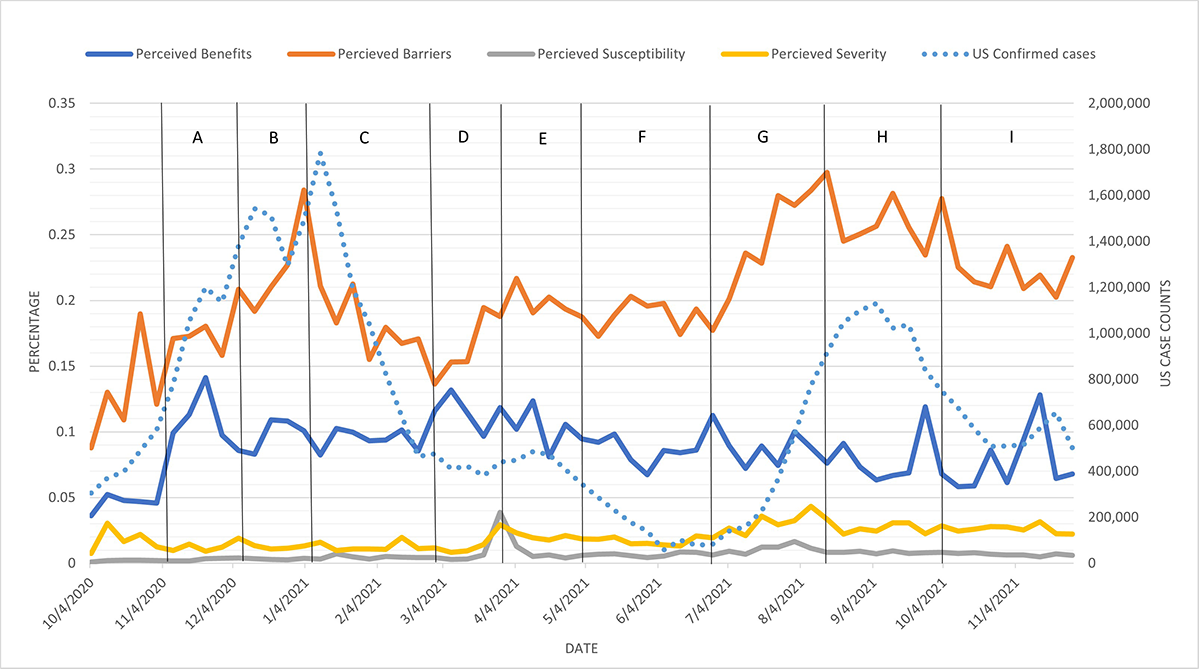
US COVID-19 case counts and each HBM scale across time for vaccine-related tweets. There was a statistically significant correlation between case counts and perceived barriers. Important events corresponding to the time intervals are listed in Table 5. HBM: Health Belief Model.

**Figure 6:**
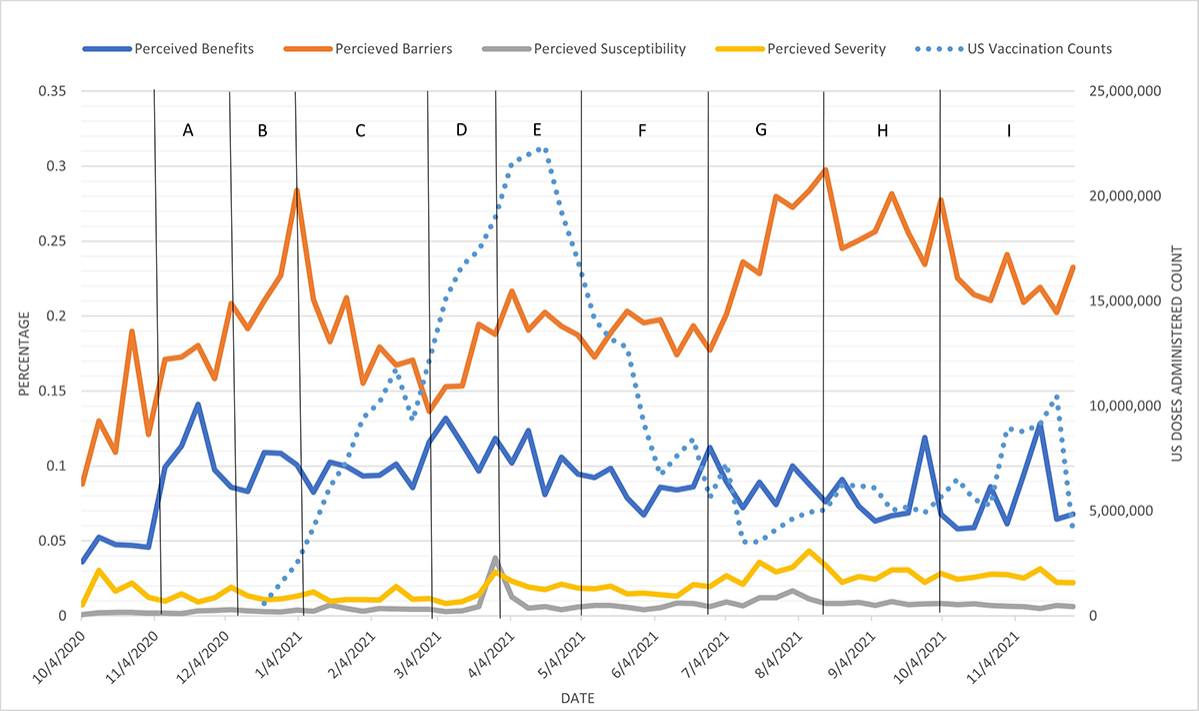
US COVID-19 vaccine doses administered and each HBM scale across time for vaccine-related tweets. There was a statistically significant negative correlation between vaccination counts and perceived barriers. Important events corresponding to the time intervals are listed in Table 5. HBM: Health Belief Model.

The HBM-positive label percentages are plotted by week in Figure 4 for mask-related tweets and Figures 5 and 6 for vaccine-related tweets. In Figures 4-6, the HBM-positive label percentages were computed by dividing raw counts of HBM labels by the total raw counts of COVID-19–related tweets filtered for mask- or vaccine-related keywords, respectively. Due to the nature of these calculations, the analysis and comparisons synthesized from Figures 4-6 focused on the direction of change in individual HBM labels rather than on the magnitude of change. Moreover, the magnitude of individual HBM label percentages were not compared across HBM label categories.

**Table 4:** Mask-related events and cues to action.

Dates		Cues to action	
**Time frame A**			
	January 21, 2020	The CDC^a^ confirms the first COVID-19 case.	
	January 31, 2020	WHO^b^ declares a global emergency.	
**Time frame B**			
	February 27, 2020	WHO issues interim guidance on masks.	
	March 8, 2020	This National Institutes of Health’s (NIH) Dr Fauci says face masks are not fully effective.	
	March 11, 2020	WHO declares a pandemic.	
	March 13, 2020	President Trump declares an emergency.	
**Time frame C**			
	April 3, 2020	The CDC recommends wearing face masks in public.	
	April 10, 2020	The first state (New Jersey) mandates face masks in public.	
	April 16, 2020	The White House begins a formal discussion to open the economy.	
	June 3, 2020	The surgeon general (HHS^c^) asks Americans to stop buying face masks.	
**Time frame D**			
	June 5, 2020	WHO recommends masks for areas with community transmission.	
	June 17, 2020	WHO halts hydroxychloroquine production.	
	July 6, 2020	WHO is asked by scientists to revise guidelines to acknowledge airborne transmission.	
	July 9, 2020	WHO declares that COVID-19 is airborne-transmissible.	
**Time frame E**			
	July 12, 2020	President Trump is seen wearing a mask in public for the first time.	
	August 13, 2020	Presidential candidate Joe Biden calls for a 3-month mask mandate.	
	August 17, 2020	The United States declares COVID-19 as the third-leading cause of death.	
	August 28, 2020	The first US reinfection case is found.	
**Time frame F**			
	September 16, 2020	President Trump releases a vaccine distribution plan.	
	September 21, 2020	The CDC withdraws guidance saying COVID-19 is airborne-transmissible.	
	October 2, 2020	President Trump and the First Lady are diagnosed with COVID-19 and the president is hospitalized.	
	October 15, 2020	WHO declares conclusive evidence that hydroxychloroquine is ineffective.	
**Time frame G**			
	November 4, 2020	The United States reports an unprecedented 100,000 cases in 1 day.	
	December 14, 2020	WHO reports the first of the COVID-19 variants (in the United Kingdom).	

^a^CDC: Centers for Disease Control and Prevention.

^b^WHO: World Health Organization.

^c^HHS: Department of Health and Human Services.

**Table 5:** Vaccine-related events and cues to action.

Dates			Cues to action
**Time frame A**			
	November 4, 2020	Joe Biden is elected the 46th President of the United States over Donald Trump.	
	November 9, 2020	Pfizer publishes vaccine results.	
	November 16, 2020	Moderna reveals vaccine efficacy results.	
**Time frame B**			
	December 4, 2020	President Biden asks Americans to commit to 100 days of wearing masks, his first act as president-elect.	
	December 11, 2020	The FDA^a^ approves the emergency use of the Pfizer vaccine.	
	December 18, 2020	The FDA approves the emergency use of the Moderna vaccine.	
	December 21, 2020	President Biden get the first dose of the vaccine.	
	December 31, 2020	The United States falls short of the goal to give 20 million vaccinations by year-end (2.8 million).	
**Time frame C**			
	January 4, 2021	The White House says more individuals will be vaccinates using reserve supplies.	
	January 6, 2021	The HHS^b^ provides US $22 billion to fund testing and vaccine distribution.	
	January 7, 2021	The CDC^c^ says COVID-19 vaccine benefits outweigh allergic reaction risks for Pfizer or Moderna.	
	January 12, 2021	The CDC and the HHS update vaccine allocation to release all available doses.	
	February 4, 2021	The FDA begins considering the J&J^d^ vaccine.	
	February 26, 2021	The Kaiser Family Foundation (KFF) poll shows vaccine acceptance increases among Americans.	
	February 27, 2021	The FDA approves the J&J vaccine with emergency use authorization.	
	March 1, 2021	The former president and First Lady urge followers to get vaccinated.	
**Time frame D**			
	March 4, 2021	The COVID-19 UK variant does not affect vaccine efficacy.	
	March 8, 2021	The CDC releases guidance on safe activities for fully vaccinated.	
	March 11, 2021	The Public Broadcasting Service (PBS) poll says nearly half of Republican men will not get COVID-19 vaccines.	
	March 11, 2021	President Biden pushes for expanded vaccine eligibility to all adults aged 18 years and older by May 1.	
	March 15, 2021	The White House unveils an expansive public relations vaccine confidence campaign.	
**Time frame E**			
	April 2, 2021	The CDC expands travel guidelines for those fully vaccinated.	
	April 6, 2021	The COVID-19 variant is detected in all 50 states.	
	April 13, 2021	The CDC and the FDA recommend pausing the J&J vaccine.	
	April 23, 2021	The CDC lifts the pause on the J&J vaccine.	
	April 27, 2021	The CDC eases mask restrictions for fully vaccinated individuals.	
**Time frame F**			
	May 4, 2021	The FDA prepares to authorize the Pfizer vaccine in adolescents.	
	May 4, 2021	President Biden announces a new goal of having 70%, or 160 million, American adults with at least 1 dose of a COVID-19 vaccine by July 4.	
	May 10, 2021	The Pfizer/BioNTech vaccine is approved for adolescents.	
	May 11, 2021	WHO^e^ declares the Delta variant a variant of concern.	
	May 24, 2021	Heart problems are investigated in vaccinated Teens.	
	June 1, 2021	Employers can require a COVID-19 vaccine.	
	June 3, 2021	The Biden administration announces a National Month of Action with the goal of immunizing at least 70% Americans by July 4.	
	June 10, 2021	President Biden is set to announce the purchase of 500 million doses of Pfizer’s COVID-19 vaccine to be donated to the rest of the world.	
	June 25, 2021	*Associated Press* reports: Almost all COVID-19 deaths recorded in the United States are among those who are not vaccinated.	
**Time frame G**			
	July 9, 2021	Pfizer says it will pursue booster shots.	
	July 12, 2021	The FDA warns that the COVID-19 J&J vaccine can lead to increased risk of Guillain-Barré syndrome.	
	July 13, 2021	HHS officials say fully vaccinated individuals do not need a COVID-19 vaccine booster shot.	
	July 21, 2021	New research published in the *New England Journal of Medicine* shows the Pfizer vaccine is not as effective against the Delta variant.	
	July 29, 2021	President Biden calls for federal worker vaccines.	
	August 3, 2021	Around 70% of Americans are vaccinated.	
**Time frame H**			
	August 12, 2021	The CDC recommends pregnant people get vaccinated.	
	August 13, 2021	A booster shot is endorsed for those immunocompromised.	
	August 18, 2021	US health officials announce a plan for booster shots for the general public.	
	August 23, 2021	The Pfizer/BioNTech vaccine gains full FDA approval.	
	August 24, 2021	Several large organizations issue vaccine mandates for workers.	
	September 9, 2021	President Biden announces all companies with over 100 employees must mandate COVID-19 vaccinations.	
	September 10, 2021	Los Angeles schools mandate vaccines.	
	September 17, 2021	The FDA committee votes against boosters for the general public.	
	September 20, 2021	Pfizer says its vaccine is safe and effective in children.	
	September 22, 2021	The FDA authorizes the Pfizer booster.	
**Time frame I**			
	October 15, 2021	The United States opens to vaccinated travelers.	
	October 25, 2021	The Moderna dose is effective in children.	
	October 29, 2021	The FDA authorizes the Pfizer vaccine for children.	
	November 1, 2021	The FDA investigates Moderna vaccine adverse effects.	
	November 5, 2021	At least 2 groups file lawsuits against implementation of President Biden’s vaccine mandate.	
	November 13, 2021	The appeals court affirms hold on the employer vaccine mandate.	
	November 19, 2021	The FDA approves a vaccine booster for all adults.	

^a^FDA: Food and Drug Administration.

^b^HHS: Department of Health and Human Services.

^c^CDC: Centers for Disease Control and Prevention.

^d^J&J: Johnson & Johnson.

^e^WHO: World Health Organization.

### Mask-Related Tweet Results

Figure 4 displays HBM label percentages. The U.S. confirmed cases by week were added to the secondary y-axis and letters within vertical lines represent a selection of events that could potentially help interpret the fluctuations in the figure from the U.S. government and related sources from the American Journal of Managed Care. Case count data came from the New York Times COVID-19 data GitHub repository [34].

Perceived benefits and perceived barriers frequently corresponded to cues for action, such as official government or public health guidance or policies (Figure 4). Perceived benefits for mask wearing trended with the rise and fall of the US COVID-19 case counts for the majority of the 2020 calendar year until mid-November and early January when vaccines were available. This observation was supported by a statistically significant (*P*<.001) Spearman correlation of 0.686. The lowest levels of perceived benefits occurred between February and mid-March, where barriers to mask wearing overshadowed benefits. Benefits first emerged above barriers after the pandemic was declared by WHO and when WHO recommended mask wearing for health care workers and sick individuals, but mask wearing was not sustained. Mask wearing emerged again more steadily when WHO declared COVID-19 was airborne-transmissible and when the CDC began recommending masks in early April. Benefits emerged most quickly after WHO changed its stance on mask wearing. The highest levels of perceived barriers were from February to mid-March. According to the top 5 retweets categorized for perceived barriers from February 24 to March 1, the “stop buying masks, save it for the health care workers” idea was the content of 4 of the top 5 most retweeted retweets. The top retweets correspond well with the initial stance of WHO regarding mask wearing for the general public [21] and the elevated perceived barriers to the action shown in Figure 4. Although WHO did not change its official stance on public mask wearing until June 5, the CDC first recommended face masks on April 3, and the first state (New Jersey) began a mask mandate on April 10. Perceived benefits gradually trended upward and peaked to correspond with each of the peaks in US case counts in late July and again in October and November and exceeded perceived barriers during much of that time. Perceived benefits trended with case counts until November around the US presidential election and in anticipation of the US Thanksgiving holiday. The lowest perceived barriers and the most significant divergence from benefits occurred as the COVID-19 case counts mounted toward their highest level in late November 2020. Table 4 lists a few of the major events and cues to action during the time frame in Figure 4.

Perceived severity and perceived susceptibility possessed a much lower percentage of HBM influence compared to perceived benefits and perceived barriers. The overall rate of perceived severity tended to be slightly higher than that of perceived susceptibility except at the beginning of the pandemic.

### Vaccine-Related Tweet Results

Figure 5 displays the HBM label percentages (raw count of HBM labels divided by the total raw count of COVID-19–related tweets filtered for vaccines). US confirmed cases by week were added to the secondary y axis, and letters within vertical lines represent a selection of events that could potentially help interpret the fluctuations in the figures from the US government and related sources from the *American Journal of Managed Care* [35,36]. In addition, US confirmed case count data were collected from the *New York Times* COVID-19 data GitHub repository [34]. Figure 6 looks at how US vaccine doses administered trended with the HBM label percentages. US vaccine data were collected from the Bloomberg Covid-19 Vaccine Tracker Open Data GitHub repository [37].

Perceived barriers and US case counts had a statistically significant (*P*=.03), positive Spearman correlation of 0.28. This means that as US case counts increased, perceived barriers also increased. Perceived barriers and US vaccination counts had a statistically significant (*P*<.001), negative Spearman correlation of –0.67. This means that there was an inverse relationship between US vaccination counts and perceived barriers. The displayed findings do not reflect magnitude but simply identify the relative volume of tweets for each of the HBM constructs.

Perceived barriers for vaccination consistently remained higher than perceived benefits, but they did not appear to trend inversely as expected. Like the face mask findings, perceived susceptibility and perceived severity accounted for a noticeably lower percentage than perceived benefits and perceived barriers. Unlike the face mask findings, perceived barriers had no crossovers with perceived benefits (Figures 5 and 6). Analysis of US case counts tended to trend with perceived barriers throughout fall 2020 through winter 2021. The percentage of perceived barriers was steadily higher than US case counts in 2021. The initial peak of perceived barriers to vaccination occurred at the beginning of the availability and distribution of vaccines. Still, it then steadily declined until early March 2021, when the CDC released guidance that safe activities were available for fully vaccinated individuals (March 8) and when President Biden pushed for expanded vaccination eligibility for all adults aged 18 years and older (March 11). The highest level of perceived barriers to vaccine acceptance peaked in late summer and early fall 2021 around the time that schools resumed operations. This rapid incline seemed to correspond with the greatest decline in US vaccination counts (Figure 6). The second greatest rise in barriers occurred early during the vaccination process from late December 2020 to early January 2021. Perceived benefits did not rise until late October to early November 2020, when vaccine efficacy was established, and then trended and held relatively steady in 2021.

Vaccination distribution rates continued to climb steadily from mid-March until mid-April 2021, but perceived barriers also continued to climb up and down. Overall vaccination counts dropped steadily in mid-April, near the time when the CDC and the Food and Drug Administration (FDA) recommended pausing the Johnson & Johnson (J&J) vaccine (April 13) and continued despite those restrictions being lifted 10 days later (April 23). The vaccination drop continued to plummet even though the FDA formally expanded the availability of vaccines for adolescents aged 12-15 years in early May (May 4). At that time, barriers again climbed upward through June 1. An inverse correlation between vaccine doses administered and perceived barriers to obtaining vaccination was observed. That is, as doses increased, perceived barriers decreased. During this inverse correlation period, WHO declared the COVID-19 Delta variant a concern (May 11), heart problems were noted in vaccinated teenagers (May 24), the government allowed employers to require the COVID-19 vaccine (June 1), and research in the *New England Journal of Medicine* indicated the Pfizer vaccine was not as effective (July 21). From June through September 2021, the conversation on perceived barriers for vaccines continued to climb, while the case counts also climbed, and doses administered declined. Table 5 lists some of the major events and cues to action during the time frame in Figures 5 and 6.

## Discussion

### Principal Findings

With COVID-19 also came the emergence of a worldwide infodemic, or the overabundance of information and misinformation about COVID-19. A central informational theme for COVID-19 in 2020 in public health revolved around controlling its spread using social distancing and face mask wearing and included ramping up vaccines that could be quickly distributed. A continuing theme in 2021 for COVID-19 involved efforts to end the pandemic through vaccine distribution and continuing some level of preventative measures (eg, stay-at-home orders, mask mandates, and capacity limits in certain businesses or other settings). However, social media played an influential role in creating an infodemic [21]. These social media influences are closely related to the political and personal reactions to COVID-19 from early in the US pandemic and appear to have continued throughout [38]. Although some social media research during the infodemic pointed to the influence of low-credibility content regarding COVID-19 [3], less has been done to understand the influence of higher-credibility content largely from prominent sources on COVID-19 health beliefs. This study used social media to investigate the COVID-19–related Twitter posts in the United States to understand health beliefs related to mask wearing and vaccinations in the midst of an infodemic. We also explored external cues to action from prominent pandemic declarations (eg, WHO and the CDC) regarding mask wearing and vaccines and notable examples of displaying preventive behaviors (eg, presidential mask wearing) and their possible association with health beliefs, as explained by the HBM constructs. Understanding the influence of social media information on COVID-19 health beliefs and preventative behaviors is important for information management during emergencies.

First, health beliefs relating to the perceived benefits of and perceived barriers to mask wearing appeared to be most influenced by external cues to action. Furthermore, they tended to inversely weakly mirror each other over time. That is, as more perceived benefits of mask wearing were discussed, fewer perceived barriers were discussed. These findings are consistent with other HBM research, particularly among student pharmacists [39]. Although previous research on COVID-19 mask-wearing beliefs pointed to the influence of perceived severity [40], in this study, the perceived susceptibility to and perceived severity of COVID-19 were much less prominent. This remained fairly consistent over time despite numerous high-profile cues to actions, such as major announcements, COVID-19 case counts, or COVID-19 deaths. This contrasts with early assumptions about what health beliefs influence face mask wearing in context with the HBM [41]. Regardless, the perceptions of disease susceptibility and severity seemed muted in this study, presumably because the worldwide pandemic may have appeared obviously relevant for most people in most places. Another possible explanation is that the immediacy of an ongoing and rapidly changing pandemic tended not to discuss the severity and seriousness of getting the disease but seemed to emphasize the importance of behaviors (ie, face masks and vaccines) and taking action.

Perceived benefits of and perceived barriers to mask wearing varied over time with sometimes dramatic swings that appeared to align with specific major cues to action. The most important or consistent cue to action was the rise and fall of total US case counts from March through December 2020 with perceived benefits. This observation is reinforced by the fact that the perceived benefits of mask wearing occurred before formal mask wearing was recommended by WHO (June 5). The benefits of mask wearing continued despite the CDC and other sources asking that masks not be hoarded so they could be available for health care workers and other individuals who were sick.

The second-most important cues to action included how the timing of certain messaging cues may have prompted temporary perceptions of the benefits of mask wearing. For example, perceived benefits peaked around the same time that WHO recommended masks in June 2020, WHO asked scientists to revise guidelines to acknowledge airborne transmission in mid-July, and President Trump was seen wearing a mask for the first time in mid-July [35]. By contrast, other kinds of messaging cues to action may have prompted a rise in perceived barriers to mask wearing when contradictory messages from WHO and the CDC urged people to not use masks early in the pandemic or messages were released about vaccines’ immediate availability at the end of 2020. Thus, the greatest cues to action for mask wearing identified from these data were primarily the rise in US case counts followed by episodic messaging that promoted the health belief that mask wearing was important. Such observed cues to action do not reflect a cause-and-effect relationship, but findings from this study are verified by what others have recently suggested [42].

Second, health belief findings regarding vaccinations from this study demonstrated several important implications for cues to action and the 4 HBM constructs. Similar to mask wearing, health beliefs relating to perceived benefits of and perceived barriers to vaccination appeared to be influenced by specific cues to action and often inversely mirrored each other over time, while not being as pronounced as mask-wearing perceptions. Twitter conversations regarding perceived vaccine barriers generally increased up until January 1 before declining and flattening. These trends tended to precede and mirror US confirmed COVID-19 case counts and death counts, suggesting the potential influence of several high-profile announcements (cues to action) during those peak times. Although previous HBM survey research in Malaysia indicated perceived barriers, such as vaccine efficacy, safety, affordability, and side effects [19], findings from this study appear to be linked more with vaccine access. Perceived barriers among COVID-19 vaccine Twitter conversations began to increase sharply when emergency use began (December 11), only peaking after the White House announced the use of reserve supplies (January 4) and the government committed funds for vaccine distribution (January 6).

Unique to vaccination Twitter conversations compared to this study’s mask-wearing findings, discussions regarding perceived benefits were always below perceived barrier trends, especially later in the pandemic. Perceived barriers reflected difficulties, challenges, conspiracies, negative effects, dangers, and perceived ineffectiveness associated with vaccinations. One potential explanation is that vaccine-related conversations became more complex because of these factors as the pandemic emerged. This complexity likely involved factors outside our study, such as political distrust, contradictory messages, and others. The Twitter conversations seemed to focus on the likelihood of the disease relative to face masks and the benefits of the behavior, but the vaccination barriers complicated the benefits of vaccination.

The number of vaccines administered peaked as perceived seriousness and perceived susceptibility discussions peaked (April 4). These findings are consistent with the HBM, which suggests behavior will change when the threat of disease increases one’s perception of seriousness and susceptibility increases. Interestingly, although the volume of discussion for perceived benefits and perceived barriers related to vaccination nearly crossed on March 4, benefit discussions remained higher than barrier discussions. Altogether, however, it appears that various cues to action beginning March 1 influenced health beliefs and ultimately vaccine behavior. This helps demonstrate that understanding the individual beliefs regarding COVID-19–preventive behaviors in response to various cues to action from these high-credibility sources is crucial toward helping manage an infodemic. Moving forward, public health officials may better manage information and positively influence health beliefs and vaccine behaviors by using traditional risk communication approaches [43]. For example, focusing on building trust through announcing early findings, being transparent with what is known and unknown, respecting public concerns, and planning in advance may serve as important lessons learned from the COVID-19 pandemic, particularly in response to vaccinations [44].

### Limitations

There are several limitations of this study. First, as noted in the study purpose justification, the results of the study are primarily exploratory in nature and should be interpreted in this context. Additional research is needed to further confirm the influence of high-profile cues to action and HBM constructs, ideally with some form of case-control or experimental design. Second, although publicly available Twitter data were used in this analysis, these data were collected from a subset of tweets returned from the Twitter Application Programming Interface (API). Because there was no way of knowing the size of the subset in relation to the whole of tweets, or the sampling method used by Twitter to create the subset, there was a potential for bias. Third, because the COVID-19–related tweets were filtered for vaccines or masks, the perceived susceptibility and perceived severity constructs may have been minimized. It may also be described by the HBM construct definitions in Tables 1 and 2, which emphasize COVID-19 itself. Regardless, filtering tweets for mask wearing and vaccines may have inadvertently diminished the susceptibility and seriousness of COVID-19 conversations with the HBM because most of these COVID-19 conversations tended to focus on the perceived benefits and perceived barriers. Fourth, future studies could use topic modeling techniques suitable for short-text documents, such as tweets, to better understand the topics surrounding masks, vaccines, and COVID-19 related to the HBM. In addition, future research might explore specifically which social media messages, communications channels, and voices are most influential on COVID-19–related prevention health beliefs. Fifth, although there are several explanations of the findings in this study, we did not attempt to establish causal relationships. Future longitudinal studies can explore questions of causation.

### Conclusion

Throughout the pandemic, experts have provided aggressive recommendations for COVID-19 prevention [21,22,24,45]. During both the COVID-19 pandemic and the infodemic, this study used a machine learning approach to explore health beliefs related to mask-wearing and vaccination recommendations and the possible influence of high-profile cues to action. Findings suggest that although certain health beliefs on Twitter appear to respond to various high-profile cues to action, health belief trends differ between mask wearing and vaccination.

## Abbreviations

AUROC: area under the receiver operating characteristic
BERT: bidirectional encoder representations from transformers
BiGRU: bidirectional gated recurrent unit
CDC: Centers for Disease Control and Prevention
DistilBERT: distilled version of bidirectional encoder representations from transformers
FDA: Food and Drug Administration
HBM: Health Belief Model
HHS: Department of Health and Human Services
J&J: Johnson & Johnson
WHO: World Health Organization
